# Endocrine diseases associated with COVID-19 vaccination: case report

**DOI:** 10.17843/rpmesp.2023.403.12572

**Published:** 2023-09-28

**Authors:** Alberto A. Teruya-Gibu, Percy Ortiz-Guerra, Abad A. Arzapalo-Poma

**Affiliations:** 1 Endocrinology service, Edgardo Rebagliati Martins National Hospital, Lima, Peru. Endocrinology service Edgardo Rebagliati Martins National Hospital Lima Peru; 2 Organ Banking Service and Histocompatibility Laboratory, Edgardo Rebagliati Martins National Hospital, Lima, Peru. Organ Banking Service and Histocompatibility Laboratory Hospital Nacional Edgardo Rebagliati Martins Lima Peru

**Keywords:** Subacute Thyroiditis, Autoimmune Hypoglycemia, COVID-19, Vaccination

## Abstract

SARS-CoV-2 vaccination is not free of adverse effects. We present two cases of endocrine involvement associated with COVID-19 vaccination. A 46-year-old woman who, after receiving the first COVID-19 vaccination dose, presented persistent fever and signs of thyrotoxicosis after being diagnosed with subacute thyroiditis associated with COVID-19 vaccination; the condition remitted with the use of corticoids. A 71-year-old male, who after COVID-19 vaccination, presented hyperinsulinemic hypoglycemia, testing positive for anti-insulin antibodies; he was diagnosed with autoimmune hypoglycemia associated with COVID-19 vaccination and received treatment with prednisone, controlling the episodes of hypoglycemia. In conclusion, endocrine diseases associated with COVID-19 vaccination are extremely rare and their timely detection allows adequate treatment.

## INTRODUCTION

SARS-CoV-2 (severe acute respiratory syndrome-Coronavirus 2) is an RNA virus from the *Coronaviridae* family. The first cases were reported in December 2019 in Wuhan, China ^1^, and in January 2020, the causative agent named COVID-19 (Coronavirus disease, 2019) was identified. On March 6, 2020, the first case was reported in Peru ^2^. The World Health Organization (WHO) declared the COVID-19 pandemic on March 11, 2020 ^3^. Since then, the Peruvian State declared a national health emergency _^(4)^_ . Vaccination began in Peru on February 9, 2021 and currently 94.05% of the adult population has received the first dose ^5^.

The safety and efficacy of vaccination against COVID-19 has been confirmed by the WHO and by some meta-analyses ^6^. Despite this, vaccination against SARS-CoV-2 is not free of adverse effects, including those affecting the endocrine system.

Moreover, some authors suggest, by studying HLA haplotypes, that genetic susceptibility may predispose to the development of events associated with infection or vaccination by COVID-19, such as immunological triggers ^7^^,^^8^.

Although the prevalence of adverse effects due to vaccination is low ^7^, it is necessary to report and identify the endocrine diseases associated with COVID-19 vaccination, in order to achieve early diagnosis and timely treatment.

We present two cases of endocrine disease associated with COVID-19 vaccination and discuss the findings in accordance with the current literature.

## CASE REPORT

### Case 1

A 46-year-old female patient, who received teleconsultation, presented general malaise two days after the first dose of Pfizer/BioNTech COVID-19 vaccine (17/07/2021), for this she was prescribed paracetamol. A week later, she presented odynophagia, sensation of a lump in the thyroid region and temperature of 38°C, for which she received ibuprofen and metamizole; fever became persistent until the day of the consultation (03/08/2021), and she also reported palpitations and tremor of the hands and feet. The laboratory test results showed C-reactive protein of 28.13 mg/L (0-5); TSH <0.007 mIU/ (normal value: 0.270 - 4.200), free T4: 35.25 pmol/L (normal value: 0.930 - 1.700), negative antithyroid antibodies and a sedimentation speed of 95 mm/h. Thyroid ultrasound (07/28/2021, Figure 1) showed hypoechogenic images with ill-defined borders in both lobes. The iodine-131 uptake test showed low uptake values at 2 and 24 hours, respectively (4.1% and 7.6%). Thyroid scintigraphy showed absence of parenchymal activity (Figure 2). Due to the persistence of fever, a nasopharyngeal swab was performed for the SARS-CoV-2 antigenic test, which was negative. Therefore, the patient was diagnosed with persistent fever due to subacute thyroiditis (SAT) associated with COVID-19 vaccine. The patient was prescribed prednisone 20 mg per day for 7 days, the fever subsided on the third day of treatment; the symptoms of thyrotoxicosis and odynophagia subsided after one week of treatment. At one month of follow-up, TSH normalized at 2.21 uIU/ml and free T4 fell slightly to 0.680 ng/dl. Thyroid function normalized after two months (TSH: 2.92; Free T4: 0.926 ng/dl). The haplotype study revealed HLA A*02/24, B*35/48, C*04/08, DR B1*14 /14, DQ A1*05/05, DQB1*03/03.

**Figure 1:**
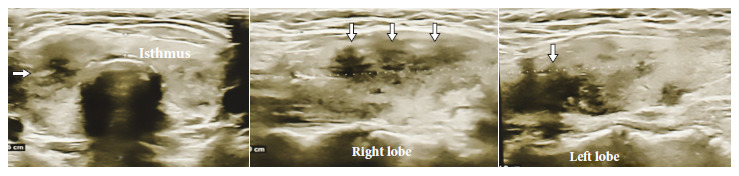
Case 1. Thyroid ultrasound showing poorly defined hypoechogenic areas (white arrows) compatible with subacute thyroiditis.

**Figure 2:**
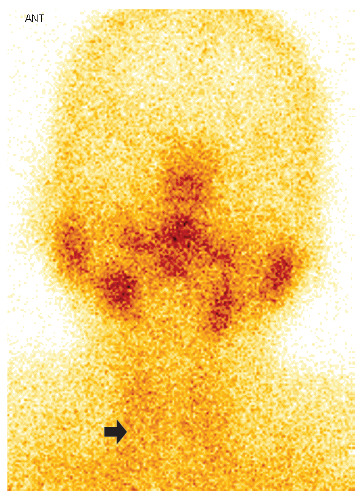
Case 1. Thyroid scan showing exclusion of thyroid silhouette (black arrow) due to subacute thyroiditis.

### Case 2

Ten days after receiving the first dose of the COVID-19 vaccine (05/05/2021), a 76-year-old male, who received teleconsultation along with his daughter, presented disorientation, stammering, and had difficulty to walk that lasted two days. Ten days after the second dose (06/13/2021), he presented severe recurrence of symptoms, associated with fasting hypoglycemia and evening postprandial hyperglycemia. The patient had a history of type 2 diabetes *mellitus* 15 years ago, arterial hypertension 20 years ago, prostate surgery, sequelae of cerebrovascular disease, left knee prosthesis and rheumatoid arthritis. He was being treated with metformin, irbesartan, clopidrogel, paracetamol, atorvastatin, tramadol, folic acid, thiamine and omeprazole. He could not be physically examined because the consultation was virtual. The patient was awake, independent, lucid, had expressive aphasia, walking difficulties, and a weight of 77 kg. Ancillary tests showed: glucose at 45 mg/dL, insulin >1000 ui/mL, C-peptide at 8.7 ng/mL, cortisol at 12.35 ug/dL, Creatinine at 0.96 mg/dL, urea at 33 mg/dL, HbA1c at 6.3%, insulin/C-peptide ratio >1, anti-insulin antibody at 106 Udes/mL (positive >18). The patient was diagnosed with autoimmune hypoglycemia associated with receiving the Pfizer-BioNTech vaccine for COVID-19. After ruling out other possible infections, the patient was prescribed prednisone 30 mg/day, which decreased progressively each month for three months without showing signs of corticoid deprivation. Fasting hypoglycemia and evening hyperglycemia remitted five months after suspending corticoid treatment. The PCR for HLA haplotype determined the HLA alleles DRB1*04/*04 and DQB1*03/03.

## DISCUSSION

Access to vaccination against SARS-CoV-2 contributed to a lower risk of severe clinical pictures after the first COVID-19 wave. These vaccines were quickly approved for use due to the rapid spread of the virus. For this reason, the reporting of adverse events associated with vaccination by COVID-19 was instrumental in documenting these cases.

Two systematic reviews ^7^^,^^8^^)^ compiled case series of endocrine diseases associated with COVID-19 vaccination, with thyroid pathology being the most frequent, as illustrated by our first case of subacute thyroiditis. Reports of SARS-CoV-2 infection cases ^9^ have shown associations between SAT and the Oxford/AstraZeneca vaccine (adenovirus vector) ^10^; the CoronaVac vaccine (inactivated SARS-CoV-2) ^11^^)^ and with the Pfizer/BioNTech vaccine (messenger RNA) ^12^. The induction mechanism could be due to an autoimmune-inflammatory syndrome induced by adjuvants ^7^^,^^8^, an induction of direct cell damage or, through antibodies against the spike protein of SARS-CoV-2, which has been shown to exhibit molecular mimicry with the thyroperoxidase protein of the thyrocyte, inducing SAT ^7^^,^^8^.

SAT pathogenesis could be linked to a genetic susceptibility associated with the HLA-B*35 haplotype in 70% of cases, likewise, other alleles such as HLA-B*18:01, -DRB1*01 and -C*04:01 have also been associated with SAT, in the present case, two susceptibility haplotypes were found, HLA-B*35 and C*04. It has been previously described that the presence of risk haplotypes can be associated with both the clinical presentation ^13^ and the characteristic ultrasound pattern of SAT ^14^. On the other hand, SARS-CoV-2, as a trigger, can act through an excessive immune response, as immunodeficiency associated with infection, or by direct cellular damage ^7^^,^^8^. Thyroid tissue shows a high expression of RCT2, particularly in women, suggesting its increased susceptibility in the development of SAT ^7^^,^^8^. Recently, an association between the HLAB*35 haplotype and susceptibility to develop SAT from vaccines has been reported ^15^. The mechanism would probably be associated with an activation of the cellular immune response ^16^ and the probable association of the HLA B35 risk haplotype with the induction of killer cells that have a cytotoxic effect ^17^.

The diagnosis of SAT in patients with COVID-19 or in those vaccinated is a challenge in clinical practice, especially if there is associated fever; therefore, quick identification can help in the management of these cases. The presence of pain in the anterior neck region, associated with systemic symptoms (malaise, fever) and symptoms of thyrotoxicosis to varying degrees (palpitations, hand tremor, irritability, fatigue and weight loss) should raise suspicion of SAT. In these cases, the request for thyroid function tests (TSH, free T4), acute phase reactants (hemogram, sedimentation rate, platelet/lymphocyte ratio, CRP) and imaging studies (thyroid ultrasound, thyroid scan and iodine uptake) can help in its detection and suggest a specific treatment based on corticosteroids and beta-blocker therapy, in order to control the systemic symptoms of this self-limited inflammatory disease ^18^. The presence of persistent fever, odynophagia and elevated acute phase reactant pointed to the described post-vaccinal symptoms or a concomitant infection with SARS-CoV-2 as differential diagnosis, being necessary to rule them out before starting corticoid treatment.

There is no previous report of autoimmune hyperinsulinemic hypoglycemia associated with COVID-19 vaccination. Autoimmune hypoglycemia (AIH) is a disease characterized by fasting hypoglycemia, elevated insulin levels (hyperinsulinemic hypoglycemia) and positive anti-insulin antibodies. Unlike other autoimmune mechanisms whose antigen is found in the tissue membrane, in AIH the antigen is insulin, which circulates in the blood ^19^. The presence of neuroglucopenic symptoms (disorientation, stammering and walking difficulty) along with glucose levels below 55 mg/dL ^19^ and elevated insulin levels are suggestive of this condition. Additionally, the presence of positive anti-insulin antibodies determined the autoimmune origin of the disease. Symptoms appeared 10 days after the first and second doses of the Pfizer-BioNTech. Fasting hypoglycemia and its resolution after food intake (Whipple’s triad) happened during the second episode, which confirmed the diagnosis; besides the patient presented postprandial hyperglycemia in the afternoon. AIH has a euglycemic phase, in which 95% of the insulin is bound to the antibody ^20^, on the other hand, during the hypoglycemic phase, free insulin increases upon release of the antibody, causing fasting hypoglycemia, as was the case in the patient. The high binding capacity of insulin to the antibody during the post-absorption phase reduces its bioavailability and can cause postprandial hyperglycemia as in the present case. AIH is strongly associated with the HLA DRB1*0406 haplotype, in these cases T cells increase when they are exposed to insulin, as has been previously described. The peptide fragment derived from the cleavage of the insulin molecule, which binds to and is presented by HLA DRB1, contains a disulfide bridge between cysteine residues in the alpha chain of the insulin molecule, which can be reduced by agents containing sulfhydryl groups. Upon cleavage of the bridge by the presence of reducing agents, insulin-derived peptides are processed by antigen-presenting cells expressing HLA DRB1*0406 and activate T helper (Th2) lymphocytes. In our case, the molecular study of the HLA haplotype determined the presence of DRB1*04 which is related to this condition in both Asian (DRB1*04:06) and non-Asian (HLADRB1*04:03) populations ^21^. Autoimmune hyperresponsiveness and molecular mimicry are the proposed mechanisms in the creation of autoantibodies and the induction of the aforementioned autoimmune diseases ^22^.

Other common autoimmune diseases of the endocrine system associated with HLA haplotype are autoimmune thyroid disease, type 1 diabetes *mellitus*, Addison’s disease and autoimmune polyglandular syndrome (APS). All of them share risk HLA haplotypes. In the case of type 2 APS, DR3-DQ2 and DRB1*04:04-DQ8 are the associated risk haplotypes, on the other hand, DR3-DQ2 and DRB1*04:01-DQ8in APS are associated with type 3. AIH can occur in isolation or accompanied by other autoimmune endocrine diseases such as Graves’ disease, in those cases, the presence of AIH could be classified as APS type 3A or 4 ^23^. In our case, the presence of the HLA DRB1*04 genotype could be considered as a criterion for APS, however, no other autoimmune endocrine conditions were identified in our patient. On the other hand, the HLA DRB1 haplotype has also been associated with rheumatoid arthritis ^24^, which was found in our patient with autoimmune hypoglycemia. Our patient with SAT had no risk representation for APS according to her HLA haplotype. On the other hand, the presence of the DQB1 haplotype in both patients could be associated with susceptibility to isolated or polyglandular autoimmune diseases that would depend on the amino acid found in position 57 of the DQB1 chain ^25^, this type of genotyping is not available in our environment.

One of the strengths of this report is that we were able to carry out follow up until remission of the endocrine involvement after treatment with corticosteroids and we were able to type the risk haplotypes described in the literature, which demonstrates the association of genetic susceptibility of these patients to COVID-19 vaccination. A limitation of this report is that the delay in diagnosis due to the availability of confirmatory results exposed both patients to continue presenting symptoms, persistent fever in the case of SAT and severe hypoglycemia in the case of AIH. Pandemic conditions and sanitary restrictions meant that care had to be provided by teleconsultation; in spite of this, it was possible to establish the diagnosis and achieve follow-up through electronic medical records and the use of social networks to maintain contact with the patients. Finally, haplotyping was performed with PCR in order to define the HLA locus and alleles. However, to determine the complete haplotyping down to proteins, a base sequencing study is required, which is not available in Peru.

The occurrence of adverse events associated with COVID-19 vaccination is extremely low (0.0042%) ^7^, so the benefit of immunization significantly exceeds the risk of these rare events that are usually treatable. However, the identification of these endocrine conditions requires a high degree of clinical suspicion for appropriate and timely management.

In conclusion, the association of endocrine diseases associated with COVID-19 vaccination are rare and occur in individuals with genetic susceptibility. Clinical suspicion is required for early diagnosis and timely treatment of these endocrine conditions.
