# Supplementary material for: Endocrine diseases associated with COVID-19 vaccination: case report
Source: Rev Peru Med Exp Salud Publica. 2023 Sep 28;40(3):364–8. doi: 10.17843/rpmesp.2023.403.12572 (PMC10953636; doi:10.17843/rpmesp.2023.403.12572)
Supplement: Translation

# Translation: Enfermedades endocrinas asociadas a vacunación por la COVID-19: reporte de casos

**Authors:** Alberto A. Teruya-Gibu, Percy Ortiz-Guerra, Abad A. Arzapalo-Poma

**Affiliations:** 1 Servicio de Endocrinología, Hospital Nacional Edgardo Rebagliati Martins, Lima, Perú.; 2 Servicio de Banco de Órganos y Laboratorio de Histocompatibilidad, Hospital Nacional Edgardo Rebagliati Martins, Lima, Perú.

**Keywords:** Tiroiditis Subaguda, Hipoglicemia Autoinmune, COVID-19, Vacunación

## Abstract

La vacunación contra el SARS-CoV-2 no está exenta de efectos adversos. Se presenta dos casos de afectación endocrina asociada a la vacunación por la COVID-19. Mujer de 46 años que, luego de la primera dosis, presentó fiebre persistente y signos de tirotoxicosis, tras el diagnóstico de tiroiditis subaguda asociada a vacunación por la COVID-19, el cuadro remitió con el uso de corticoides. Varón de 71 años, que luego de la vacunación por la COVID-19, presentó hipoglicemias hiperinsulinemicas, con resultado positivo de anticuerpos antiinsulina. Se le diagnosticó con una hipoglicemia autoinmune asociada a la vacunación por la COVID-19 y recibió tratamiento con prednisona, controlando los episodios de hipoglicemia. En conclusión, las enfermedades endocrinas asociadas a vacunación por la COVID-19 son extremadamente raras y su detección oportuna permite su tratamiento adecuado.

## INTRODUCCIÓN

SARS-Cov-2 (*Severe Acute Respiratory Syndrome-Coronavirus 2*) es un virus ARN perteneciente a la familia de los *Coronaviridae*. Los primeros casos se reportaron en diciembre del 2019 en Wuhan, China ^1^ y en enero del 2020, se identificó el agente causal denominado COVID-19 (*Coronavirus disease*, 2019). El 6 de marzo del 2020 se reportó el primer caso en el Perú ^2^. La pandemia de la COVID-19 fue declarada por la Organización Mundial de la Salud (OMS) el 11 de marzo del 2020 ^3^. Desde esa fecha, el Estado Peruano declaró la emergencia sanitaria a nivel nacional ^4^. La vacunación se inició en Perú el 9 de febrero del 2021 y actualmente el 94,05% de la población adulta ha recibido su primera dosis ^5^.

La seguridad y eficacia de la vacunación contra la COVID-19 ha sido confirmada por la OMS y por algunos metaanálisis ^6^. A pesar de ello, la vacunación contra el SARS-CoV-2 no está exenta de efectos adversos, entre ellos, las que afectan al sistema endocrino.

Más aún, algunos estudios sugieren que existiría una susceptibilidad genética demostrable mediante el estudio de haplotipos HLA que predispone al desarrollo de eventos asociados a infección o vacunación por la COVID-19, como gatillos inmunológicos ^7^^,^^8^.

Si bien, la prevalencia de efectos adversos por la vacunación es baja ^7^, es necesario reportar y conocer las patologías endocrinas que se asocian a la vacunación por la COVID-19, lo que permitirá un reconocimiento temprano para un tratamiento oportuno.

En el presente reporte, se describen dos casos de afectación endocrina asociada a la vacunación por la COVID-19 y se discuten los hallazgos de acuerdo con la literatura revisada. 

## REPORTE DE CASOS

### Caso 1

Paciente mujer de 46 años; que acudió a teleconsulta por presentar, dos días posterior a la primera dosis de la vacuna Pfizer/BioNTech para la COVID-19 (17/07/2021), malestar general, motivo por el que tomó paracetamol; a la semana se agrega odinofagia y sensación de bulto en la región tiroidea y fiebre cuantificada en 38°C, motivo por el que tomó ibuprofeno y metamizol, la fiebre se volvió persistente hasta el día de la consulta (03/08/2021), refiriendo, además, palpitaciones, temblor de manos y pies. En los análisis realizados se encontraron: proteína C reactiva en 28,13 mg/L (0-5); TSH <0,007 mUI/ (valor normal: 0.270 - 4.200), T4 libre 35,25 pmol/L (valor normal: 0.930 - 1.700), anticuerpos antitiroideos negativos y velocidad de sedimentación en 95 mm/h. En la ecografía de tiroides (28/07/2021, Figura 1) se evidenció imágenes hipoecogénicas de bordes mal definidos en ambos lóbulos. Se realizó una captación de I-131, encontrándose valores de captación bajas a las dos y 24 horas, respectivamente (4,1% y 7,6%). En la gammagrafía tiroidea se evidenció ausencia de actividad parenquimal (Figura 2). Por la persistencia de la fiebre, se le realizó un hisopado nasofaríngeo para la prueba antigénica de SARS- CoV-2, con resultado negativo. Con los hallazgos descritos se definió el diagnóstico de fiebre persistente por tiroiditis subaguda (TSA) asociada a vacuna para la COVID-19. Se le indicó prednisona 20 mg día por 7 días, remitiendo la fiebre al tercer día de iniciado tratamiento; la sintomatología de tirotoxicosis y odinofagia remitieron a la semana de tratamiento. Al mes de control se mostró normalización de TSH en 2,21 uUI/ml y leve caída de la T4 libre a 0,680 ng/dl. A los dos meses se normalizó la función tiroidea (TSH: 2,92; T4 Libre: 0,926 ng/dl). El estudio de haplotipo reveló HLA A*02/24, B*35/48, C*04/08, DR B1*14 /14, DQ A1*05/05, DQB1*03/03.

**Figura 1:** Caso 1. Ecografía tiroidea que muestra áreas hipoecogénicas mal delimitadas (flechas blancas) compatibles con tiroiditis subaguda.

**Figura 2:** Caso 1. Gammagrafía tiroidea donde se muestra exclusión de silueta tiroidea (fecha negra) por la tiroiditis subaguda.

### Caso 2

Varón de 76 años, que acudió a teleconsulta con su hija por presentar, 10 días posterior a la primera dosis de la vacuna para la COVID-19 (05/05/2021), desorientación, balbuceo, dificultad para deambular que duró dos días. Luego de 10 días de la segunda dosis (13/06/2021), presentó recurrencia severa de los síntomas, asociado a hipoglicemia en ayunas e hiperglicemia posprandial vespertina. El paciente tenía antecedente de diabetes *mellitus* tipo 2 hace 15 años, hipertensión arterial hace 20 años, cirugía prostática, secuela de enfermedad cerebrovascular, prótesis de rodilla izquierda y artritis reumatoidea. Recibía tratamiento con metformina, irbesartan, clopidrogel, paracetamol, atorvastatina, tramadol, ácido fólico, tiamina y omeprazol. El examen físico fue diferido por ser atención de teleconsulta, se observó un paciente despierto, con afasia de expresión, se consigna en la historia clínica electrónica un peso de 77 Kg, limitación funcional para caminar, independiente y lúcido. En los exámenes auxiliares se encontró: glucosa en 45 mg/dL, insulina >1000 ui/mL, péptido C en 8,7 ng/mL, cortisol en 12,35 ug/dL, Creatinina en 0,96 mg/dL, urea en 33 mg/dL, HbA1c en 6,3%, ratio insulina/péptido C >1, Anticuerpo antiinsulina en 106 Udes/mL (positivo >18). Se realizó el diagnóstico de hipoglicemia autoinmune asociada a la vacunación con Pfizer-BioNTech para la COVID-19. Previo descarte de probables focos infecciosos, se le indicó prednisona 30 mg/día con disminución progresiva de la dosis mensual durante tres meses, sin mostrar deprivación por corticoides; luego de cinco meses de haber suspendido el corticoide, se confirmó remisión de la hipoglicemia en ayunas y de la hiperglicemia vespertina. El estudio molecular por PCR para haplotipo HLA determinó los alelos HLA DRB1*04/*04 y DQB1*03/03.

## DISCUSIÓN

Luego de la primera ola de la COVID-19, el acceso a la vacunación contra el SARS-CoV-2 ha contribuido con un menor riesgo de cuadros clínicos severos. Debido a la propagación rápida del virus, los procesos de aprobación para el uso de estas vacunas fueron aceleradas por la emergencia sanitaria. Por este motivo, la notificación de eventos adversos asociados a la vacunación por la COVID-19 fue determinante para documentar estos casos en múltiples órganos y sistemas, entre ellos el sistema endocrino.

Dos revisiones sistemáticas ^7^^,^^8^ han compilado series de casos de patología endocrina asociada a la vacunación por la COVID-19, siendo la patología tiroidea la más frecuente, como lo ilustra nuestro primer caso de tiroiditis subaguda. En casos de infección por el SARS-CoV-2 ^9^, se han reportado TSA asociada a la vacunación con Oxford/AstraZeneca, que contiene un vector de adenovirus ^10^; con CoronaVac, que contiene SARS-CoV-2 virus inactivado ^11^ y con la vacuna ARN mensajero Pfizer/BioNTech ^12^. El mecanismo de inducción podría deberse a un síndrome autoinmune-inflamatorio inducido por adyuvantes ^7^^,^^8^, una inducción de daño celular directo o, a través de los anticuerpos contra la proteína *spike* de SARS-CoV-2, que ha demostrado presentar mimetismo molecular con la proteína tiroperoxidasa del tirocito, induciéndose la TSA ^7^^,^^8^.

La patogénesis de TSA estaría vinculado a una suceptibilidad genética asociada al haplotipo HLA-B*35 en el 70% de los casos, asimismo, otros alelos como HLA-B*18:01, -DRB1*01 y -C*04:01 también se han asociado a TSA, en el presente caso se encontraron dos haplotipos de suceptibilidad, HLA-B*35 y C*04. Se ha descrito que la presencia de haplotipos de riesgo se puede asociar tanto a la forma de presentación clínica ^13^ como al patrón ecográfico característico de TSA ^14^. Por su lado, el SARS-CoV-2, como gatillo, puede actuar a través de una excesiva respuesta inmune, inmunodeficiencia asociada con infección, o daño celular directo ^7^^,^^8^. El tejido tiroideo muestra una gran expresión de ECA2, especialmente en mujeres, lo que sugiere su mayor susceptibilidad en el desarrollo de TSA ^7^^,^^8^. Últimamente, se ha descrito una asociación entre el haplotipo HLAB*35 y la susceptibilidad a presentar TSA por vacunas ^15^. El mecanismo probablemente se asociaría a una activación de la respuesta inmune celular ^16^ y la probable asociación del haplotipo de riesgo HLA B35 con la inducción de células asesinas que tienen efecto citotóxico ^17^.

El diagnóstico de TSA en pacientes con infección o vacunación por la COVID-19 constituye un reto en la práctica clínica, sobre todo si hay fiebre asociada, por lo que su reconocimiento puede ayudar a su manejo específico en estos casos. La presencia de dolor en la región anterior del cuello, asociado a síntomas sistémicos (malestar general, fiebre) y síntomas de tirotoxicosis en diversos grados (palpitaciones, tremor de manos, irritabilidad, cansancio y baja de peso) deben hacernos sospechar en TSA. En estos casos, la solicitud de pruebas de función tiroidea (TSH, T4 libre), reactantes de fase aguda (hemograma, velocidad de sedimentación, índice plaquetas/linfocitos, PCR) y estudios de imágenes (ecografía tiroidea, gammagrafía tiroidea y captación de yodo) pueden ayudar a su detección y plantear un tratamiento específico basado en terapia con corticoides y betabloqueadores, a fin de controlar los síntomas sistémicos de esta enfermedad inflamatoria autolimitada ^18^. La presencia de fiebre persistente, odinofagia y reactante de fase aguda elevada planteaban como diagnóstico diferencial los síntomas posvacunales descritos o una infección concomitante con SARS-CoV-2, siendo necesario su descarte antes de iniciar el tratamiento con corticoides.

Nuestro caso de hipoglicemia hiperinsulinémica autoinmune asociada a la vacunación por la COVID-19 no está descrita en la literatura. La hipoglicemia autoinmune (HAI) es una enfermedad caracterizada por hipoglicemia en ayunas, niveles elevados de insulina (hipoglicemia hiperinsulinémica) y anticuerpos antiinsulina positivos. A diferencia de otros mecanismos autoinmunes cuyo antígeno se encuentra en la membrana de los tejidos, en la HAI el antígeno es la insulina, que circula en sangre ^19^. La presencia de síntomas neuroglucopénicos (desorientación, balbuceo y dificultad para deambular) con niveles de glucosa menores a 55 mg/dL ^19^ y niveles elevados de insulina sugieren esta entidad. Adicionalmente, la presencia de anticuerpos antiinsulina positivos determinó el origen autoinmune de la enfermedad. La sintomatología se presentó a los 10 días de la primera y segunda dosis de la vacuna Pfizer-BioNTech, siendo en el segundo episodio donde se corrobora la hipoglicemia en ayunas y su resolución tras la ingesta de alimentos (tríada de Whipple); asimismo, el paciente presentaba hiperglicemia posprandial vespertina. En la HAI, se describe una fase euglicémica, en la que el 95% de la insulina se encuentra unida al anticuerpo ^20^, mientras que, en la fase hipoglucémica, la insulina libre se encuentra incrementada al liberarse del anticuerpo, ocasionado las hipoglicemias en ayunas, como lo que ocurría en el paciente. Durante la fase posterior a la absorción de los alimentos, la alta capacidad de unión de la insulina con el anticuerpo reduce su biodisponibilidad y puede ocasionar hiperglicemias posprandiales como en el presente caso. La HAI se asocia fuertemente con el haplotipo HLA DRB1*0406, en estos casos se ha descrito una proliferación incrementada de células T, cuando estas células son expuestas a la insulina. El fragmento peptídico derivado del clivaje de la molécula de insulina, que se une y es presentado por HLA DRB1, contiene un puente disulfuro entre residuos de cisteína en la cadena alfa de la molécula de insulina, el cual puede ser reducido por agentes que contienen grupos sulfhidrilos. Al clivarse el puente por la presencia de agentes reductores, los péptidos derivados de la insulina son procesados por las células presentadoras de antígenos que expresan HLA DRB1*0406 y activan a los linfocitos T helper (Th2). En nuestro caso, el estudio molecular del haplotipo HLA determinó la presencia de DRB1*04 que se encuentra relacionado a esta patología, tanto en población asiática (DRB1*04:06) como no asiática (HLADRB1*04:03) ^21^. La hiperrespuesta autoinmune y el mimetismo molecular, son los mecanismos propuestos en la generación de autoanticuerpos y la inducción de las mencionadas enfermedades autoinmunes ^22^.

Otras enfermedades autoinmunes frecuentes del sistema endocrino asociadas a haplotipo HLA son la enfermedad tiroidea autoinmune, diabetes *mellitus* tipo 1, enfermedad de Addison y el síndrome poliglandular autoinmune (SPA). Todas ellas comparten haplotipos HLA de riesgo. En el caso del SPA tipo 2 los haplotipos de riesgo asociados son DR3-DQ2 y DRB1*04:04-DQ8, mientras que en el SPA tipo 3 se asocia a DR3-DQ2 y DRB1*04:01-DQ8. La HAI puede producirse en forma aislada o acompañada de otras enfermedades endocrinas autoinmune como la enfermedad de Graves, en esos casos, la presencia de HAI podría ser clasificada como un SPA tipo 3A o 4 ^23^. La presencia del genotipo HLA DRB1*04 en nuestro caso podría ser considerada como criterio para SPA, sin embargo, no se identificaron otras patologías endocrinas autoinmunes en nuestro paciente. Por otra parte, el haplotipo HLA DRB1 también se ha asociado a artritis reumatoide ^24^, el cual sí se encontraba como antecedente en nuestro paciente con hipoglicemia autoinmune. En nuestra paciente con TSA, no tuvo representación de riesgo para SPA según su haplotipo HLA. Por otro lado, la presencia del haplotipo DQB1 en ambos pacientes podría estar asociada a una susceptibilidad de tener enfermedades autoinmunes aisladas o poliglandulares que dependerían del aminoácido se encuentre en la posición 57 de la cadena DQB1 ^25^, este tipo de genotipificación no está disponible en nuestro medio.

Como fortaleza del presente reporte, se debe mencionar que se logró hacer un seguimiento hasta la remisión de la afectación endocrina tras el tratamiento con corticoides y se logró tipificar los haplotipos de riesgo descritos en la literatura que demuestra la asociación de susceptibilidad genética de estos pacientes ante la vacunación por la COVID-19. Como limitaciones, el retraso en el diagnóstico en función de la disponibilidad de los resultados confirmatorios expuso a ambos pacientes a continuar con sus síntomas, fiebre persistente en el caso de la TSA e hipoglicemias severas en el caso de la HAI. Las condiciones de pandemia y las restricciones sanitarias obligaron a que las atenciones se realizaran por teleconsulta; a pesar de ello, se logró plantear el diagnóstico y lograr el seguimiento a través de la historia clínica electrónica y el uso de redes sociales para mantener el contacto con los pacientes. Finalmente, la tipificación de haplotipos se efectuó con PCR para definir los locus y alelos HLA. Sin embargo, para determinar el haplotipaje completo hasta proteínas se requiere un estudio de secuenciamiento de bases, el cual no se realiza en el país.

La presentación de eventos adversos asociados a vacunación por COVID-19 resulta extremadamente baja, 0,0042% ^7^, por lo que el beneficio de la inmunización excede en forma importante el riesgo de estos eventos raros que suelen ser tratables. Sin embargo, la identificación de estas patologías endocrinas requiere un alto grado de sospecha clínica para un manejo apropiado y oportuno.

En conclusión, la asociación de enfermedades endocrinas asociadas a la vacunación por la COVID-19 son raras y se presentan en personas con susceptibilidad genética. Se requiere alta sospecha clínica para un diagnóstico temprano y tratamiento oportuno de estas afecciones endocrinas.
